# HIV-1 cell-to-cell transmission and broadly neutralizing antibodies

**DOI:** 10.1186/s12977-018-0434-1

**Published:** 2018-07-28

**Authors:** Jérémy Dufloo, Timothée Bruel, Olivier Schwartz

**Affiliations:** 1 Virus and Immunity Unit, Department of Virology, Institut Pasteur, Paris, France; 2 CNRS-UMR3569, Paris, France; 3 Vaccine Research Institute, Créteil, France

**Keywords:** HIV-1, bNAbs, Cell-to-cell transmission, Neutralization

## Abstract

HIV-1 spreads through contacts between infected and target cells. Polarized viral budding at the contact site forms the virological synapse. Additional cellular processes, such as nanotubes, filopodia, virus accumulation in endocytic or phagocytic compartments promote efficient viral propagation. Cell-to-cell transmission allows immune evasion and likely contributes to HIV-1 spread in vivo. Anti-HIV-1 broadly neutralizing antibodies (bNAbs) defeat the majority of circulating viral strains by binding to the viral envelope glycoprotein (Env). Several bNAbs have entered clinical evaluation during the last years. It is thus important to understand their mechanism of action and to determine how they interact with infected cells. In experimental models, HIV-1 cell-to-cell transmission is sensitive to neutralization, but the effect of antibodies is often less marked than during cell-free infection. This may be due to differences in the conformation or accessibility of Env at the surface of virions and cells. In this review, we summarize the current knowledge on HIV-1 cell-to-cell transmission and discuss the role of bNAbs during this process.

## Background

Human Immunodeficiency Virus (HIV-1) is the etiological agent of AIDS [1]. Identification of molecular mechanisms governing the replication of HIV-1 allowed the design of potent antiretroviral treatment (ART). Combined ART restored the life expectancy of patients, transforming a fatal infection into a manageable chronic disease. However, limited access to therapy in many regions of the world and the existence of a viral reservoir insensitive to treatment urge the need for novel antiviral strategies.

HIV-1 infects cells by multiple mechanisms, either as cell-free or cell-associated particles [2, 3]. HIV-1 infection is more efficient when the virus is transmitted through direct cell contacts. HIV-1 follows different routes of cell-to-cell transmission [4]. One main mechanism involves a structure called the Virological Synapse (VS). It allows the polarized delivery of newly formed viral particles [5, 6]. Its organization requires both cellular and viral proteins. The virus also hijacks other cellular pathways to spread, such as nanotubes, filopodia, phagocytic or endocytic compartments.

As part of the immune response, infected individuals rapidly develop anti-HIV-1 antibodies, as soon as one week following initial viral exposure [7]. These early-produced antibodies do not neutralize the virus [7]. The first neutralizing antibodies are detected two to three months later [8]. These antibodies are inefficient against heterologous viral strains and are rapidly escaped by mutation of the autologous virus [9, 10]. Some patients called elite neutralizers develop antibodies with broad neutralization potency [11]. Deconvolution of their polyclonal response enabled the identification of several monoclonal bNAbs (reviewed in [12]). Potent bNAbs present peculiar molecular features, such as intensive hypermutation and often long CDRH3 regions (reviewed in [13]). bNAbs target conserved regions on the viral Env spike, called sites of vulnerability [13]. These include the CD4 binding site (CD4bs), the N-glycans of V1/V2 and V3 loops, the gp41 membrane proximal external region (MPER), and the gp120/gp41 interface, which comprises a recently described epitope composed of the fusion peptide at the N-terminus of gp41 and the N88 glycan on gp120 [14–16]. bNAbs are often screened and selected with assays that use cell-free virus. The capacity of bNAbs to suppress cell-to-cell transmission has been thus often under-evaluated. In vitro, bNAbs neutralize cell-free infection by many viral strains and trigger Fc-mediated effector mechanisms, including antibody-dependent cellular cytotoxicity (ADCC) [17]. In animal models, bNAbs display both prophylactic [18] and therapeutic efficacy (reviewed in [19]). They clear HIV-infected cells and modulate host immune responses [20, 21]. These findings suggest that bNAbs could target the latent HIV reservoir and contribute to long-term remission of HIV-1 infection in humans.

Phase 1 studies of bNAbs targeting the CD4bs (3BNC117 and VRC01) and the V3 loop (10-1074) demonstrated their safety and efficacy (reviewed in [22]). Infusion of single bNAbs induced a transient decline in viremia of approximately 1.5 log_10_ copies/ml, followed by selection of escape variants [23–25]. Of note, the half-life of 10-1074 (24 days) was higher than that of 3BNC117 and VRC01 (around 15 days). In ART-treated patients pre-screened for their susceptibility to 3BNC117, infusion of this antibody delayed viral rebound after ART cessation by an average of 8 weeks [26]. Moreover, 3BNC117 potentiated subsequent anti-HIV-1 host antibody responses, demonstrating an immunomodulatory potential that is not fully understood [27]. Thus, understanding the molecular and cellular bases of bNAbs antiviral activity is critical to optimize their in vivo efficacy.

In this review, we first summarize the current knowledge on HIV cell-to-cell transmission. We discuss the mechanisms that may account for the differences observed in neutralization of cell-free and cell-associated HIV-1. We then detail how bNAbs bound at the cell surface neutralize viral propagation but also destroy infected cells by ADCC and other mechanisms.

## HIV-1 cell-to-cell transmission

### The virological synapse between infected an uninfected T cells

**Fig. 1:**
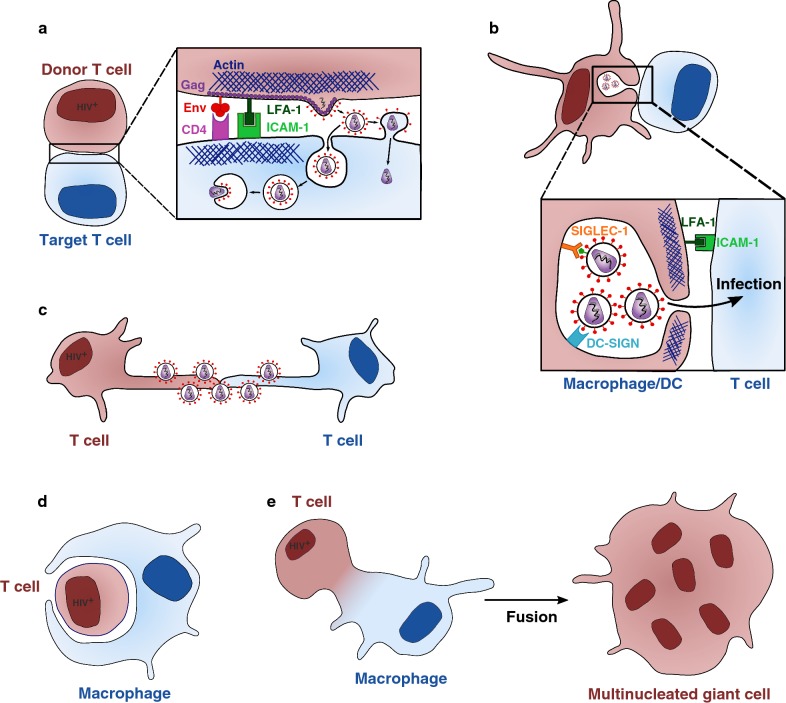
Mechanisms of HIV-1 cell-to-cell transmission. **a** Infected and uninfected T cells come in contact to form a virological synapse. HIV-1 gains access to the cytoplasm of the target cell by direct fusion at the plasma membrane or eventually after endocytosis. This structure is dependent on Env/CD4 interaction, adhesion molecules (LFA-1/ICAM-1) interaction, and the cytoskeleton. **b** Uninfected macrophages or dendritic cells (DC) store HIV-1 particles in intracellular compartments after capture via DC-SIGN or SIGLEC-1. These particles can be released and transferred to CD4^+^ T cells through the infectious synapse. **c** HIV-1 surfs along nanotubes between uninfected and infected T cells. **d** Macrophages can be infected after phagocytosis of infected CD4^+^ T cells. **e** Macrophages can fuse with infected CD4^+^ T cells and with surrounding uninfected macrophages to form multinucleated giant cells. Donor cells are in brown and uninfected cells in blue

Early studies reported secretion of HIV-1 particles and relocalization of adhesion molecules at the contact zone between infected and uninfected T cells [28–30]. The precise mechanisms of viral cell-to-cell transmission were initially described with another retrovirus, Human T cell Leukemia Virus type 1 (HTLV-I) [31]. Upon cell–cell contacts, HTLV-I Env, Gag and the viral genome accumulate at cell–cell junctions, allowing polarized budding of viral particles and their transfer to the target cells in a confined area. Igakura and colleagues named this structure the “virological synapse” (VS) due to its similarities with the immunological synapse that forms between T lymphocytes and Dendritic Cells (DCs) during antigen presentation [32]. The VS was then observed during HIV-1 spread in T cells [5]. The HIV-1 VS displayed similar features: recruitment of Env and Gag at the interface on the producer cell side and of the cytoskeleton on the target side [5] (Fig. 1a). An infected cell can generate more than one VS, allowing simultaneous transfer of HIV-1 to multiple targets [33].

HIV-1 drives the organization of the VS. The VS is initiated by interactions between Env on the donor and CD4 on the target cell [5]. Env-mediated fusion seems to be regulated at the VS to decrease or slow down the formation of syncytia. The interaction between the Env cytoplasmic domain and the underlying immature Gag (p55) lattice reduces Env fusogenicity [34]. Fusion is also impaired by cellular proteins, such as tetraspanins or ezrin that accumulate at the VS [35, 36]. Co-receptor (CCR5 or CXCR4) engagement is not necessary for VS formation and transfer of virions [37]. However, co-receptors are required for subsequent productive infection [38].

After initial CD4/Env interactions, cellular adhesion molecules such as LFA-1, ICAM-1 and ICAM-3 are recruited to stabilize the VS [33, 39]. These adhesins are not mandatory, as blocking ICAM-1/3 and LFA-1 by antibodies does not inhibit the creation of cell conjugates and viral transfer [40]. Whether the recruitment of adhesion molecules to the VS is involved in its stabilization or has other functions is not fully understood. The cytoskeleton plays a predominant role during HIV-1 cell-to-cell transmission. The formation of the VS depends on actin and tubulin [5, 41, 42], and is associated with a relocalization of the MTOC towards the site of cell–cell contact, which contributes to the trafficking of viral and cellular proteins to the VS [31, 43, 44]. However, viral transfer can occur simultaneously to multiple targets, even if the MTOC is localized towards a single recipient cell [43]. Lipid rafts also promote Gag and Env clustering at the synapse [45].

Various viral and cellular proteins modulate positively or negatively HIV-1 cell-to-cell transfer. The viral protein Nef promotes the accumulation of Gag below the cellular membrane, increasing the transfer of mature HIV-1 virions and productive infection of target cells [46]. BST2/Tetherin, an interferon-induced gene that restricts HIV-1, accumulates with Gag and actin at the VS in infected donor cells and limits viral cell-to-cell spread [47, 48]. However, the inhibitory effect of tetherin is debated [49]. IFITM3, another interferon-stimulated gene with antiviral activities, also impairs cell-to-cell transfer of HIV-1 when expressed on either donor and target cells [50] and may act by infiltrating budding viral particles [50, 51].

Following VS formation, depending on the cell types used, newly produced viral particles can either fuse directly at the target cell plasma membrane [5] or be endocytosed by the acceptor T cell in a clathrin- and dynamin-dependent manner [38, 52, 53] (Fig. 1a). It has been proposed that HIV-1 viral particles transferred through the VS may undergo maturation after endocytosis [54]. However, this route of entry has not been observed during cell-free infection [55]. Whatever the entry route, polarized HIV-1 budding leads to a massive release of viral particles into the cytoplasm of the target cell. This high multiplicity of infection (MOI) leads to a two to three log increase in the efficiency of transmission for cell-associated HIV-1 compared to cell-free virus [37, 56, 57]. It also enhances the number of integrated proviruses [58, 59], and accelerates viral gene expression and spread [60].

VS formation has been observed in vivo by intravital imaging of mice infected with the Friend murine leukemia virus [61]. This study confirmed the role of Env for VS formation and the polarization of Gag at the sites of cell–cell contact in vivo. HIV-1 spread has also been studied in humanized mouse models. HIV-1-infected T cells migrate to lymph nodes, where they bind to target cells, transfer the virus, and also form syncytia [62, 63]. Tomographic analyses identified HIV-1 budding at sites of close cell–cell contact through LFA-1- and ICAM-1-positive structures [64]. Furthermore, the observation of Env-dependent stable contacts between infected and uninfected CD4^+^ T cells, co-transmission of multiple viral genotypes, and foci of viral replication suggests that cell-to-cell transmission occurs in lymphoid organs of humanized mice [65].

Thus, T cell-to-T cell transmission of HIV-1 is highly efficient in vitro and likely contributes to viral dissemination in vivo.

### The infectious synapse between DCs/macrophages and T cells

DCs and macrophages transmit HIV-1 to T cells through different routes, namely *cis*- and *trans*-infections. During *cis*-infection, DCs and macrophages are productively infected and transmit HIV-1 to CD4^+^ T cells through a VS-like structure [66–68]. However, DCs are relatively resistant to productive infection [69]. They express SAMHD1, which inhibits reverse transcription [70–72] and regulates immune sensing and host responses [73–75]. These cells express low levels of HIV-1 receptor and co-receptors [76, 77]. Macrophages can be productively infected by HIV-1, which buds and accumulates into intracellular tetraspanin-rich compartments termed Virus Containing Compartments (VCCs) [78–80]. VCCs are connected to the cell membrane and release virus to neighboring cells [80].

DCs or macrophages that have captured viral particles but are not productively infected also transmit HIV-1 to CD4^+^ T cells [81]. This *trans*-infection mechanism is thought to play a role in vivo (reviewed in [82]). DCs and macrophages may capture HIV-1 in a CD4-independent manner. Different cellular proteins bind HIV-1 particles. Env interacts with the C-type lectin DC-SIGN prior to internalization into VCCs [83–86]. In mature DCs, Siglec-1 capture virions in an Env-independent manner by binding to gangliosides present on the viral membrane, also leading to internalization into VCCs [87–90]. After capture, HIV-1 is transferred to T cells through a structure reminiscent of the VS: the Infectious Synapse (IS) [91] (Fig. 1b). In contrast to the VS, CD4 and Env are dispensable for the formation of the IS, but are necessary for viral fusion and productive infection of T cells [92]. IS formation and subsequent viral transfer require the cortical actin cytoskeleton, which is stabilized by tetraspanin-7 and dynamin-2 in DCs [93]. Interactions between LFA-1 and ICAM-1, and between MHC and TCR modulate DC-to-T cell *trans*-infection [91]. Exosomes released by DCs may also facilitate viral transfer [94]. Recent multidimensional techniques have revealed that the myeloid compartment is more complex than initially thought and comprises at least four monocyte and six DC subsets, including novel pre-DC and plasmacytoid DC (pDC) populations [95, 96]. It will be of interest to determine the sensitivity to HIV-1 infection and the ability to transfer the virus across the spectrum of DC subsets [97].

### Other modes of cell-to-cell transmission of HIV-1

Various additional modes of cell-associated HIV-1 transfer have been reported. HIV-1 can use close-ended membrane protrusions called tunneling nanotubes (TNTs) that form between infected and uninfected T cells to spread in a receptor-dependent manner [98] (Fig. 1c). A similar usage of TNTs was observed in macrophages and it has been proposed that Nef induces these TNTs [99, 100]. HIV-1 is contained within endosomes during TNT-mediated transfer in macrophages [101, 102]. Actin-rich membrane protrusions called filopodia are also induced in DCs after interaction between HIV-1 and DC-SIGN, in a Cdc42- [103] and Diaph2-dependent manner [93, 104], facilitating HIV-1 transfer to CD4^+^ T cells.

Macrophages also engulf living or dying HIV-1-infected T cells allowing their productive infection [105] (Fig. 1d). The impact of the most potent bNAbs on this mode of transmission has not been assessed yet. A two-step process for transfer of HIV-1 from infected CD4^+^ T cells to macrophages has been described [106]. First, CD4^+^ T cells establish a contact with macrophages and fuse. This macrophage-T cell hybrid will then fuse with surrounding uninfected macrophages, spreading the infection via multinucleated giant cells (Fig. 1e). CD4^+^ T cells can also form a VS-like structure with epithelial cells from the genital mucosa, which leads to transcytosis of HIV-1 through the epithelium and subsequent infection of stromal macrophages [107, 108].

Overall, HIV-1 hijacks various pathways to spread across cells that contact each other. This likely contributes to inter- and intra-individual viral propagation. Thus, efficacious antiviral agents must block both cell-free and cell-to-cell infection.

## bNAbs and cell-to-cell transmission of HIV-1

### Inhibition of HIV-1 transmission through the virological synapse

Before the discovery of bNAbs, several studies investigated the capacity of antibodies to block HIV-1 cell-to-cell transmission. Some sera from infected patients lost their neutralizing activity when the source of HIV-1 was cell-associated [57, 109, 110]. The ability of patients’ sera to maintain activity against cell-associated HIV-1 was patient-dependent and correlated with the neutralization breadth [111]. First generation neutralizing monoclonal antibodies, such as the anti-MPER 2F5 and 4E10, the anti-V3 antibody 257-D, and the anti-CD4bs b12 were also tested in cell–cell assays, but results were conflicting, and no clear pattern could be determined [112–114].

**Fig. 2:**
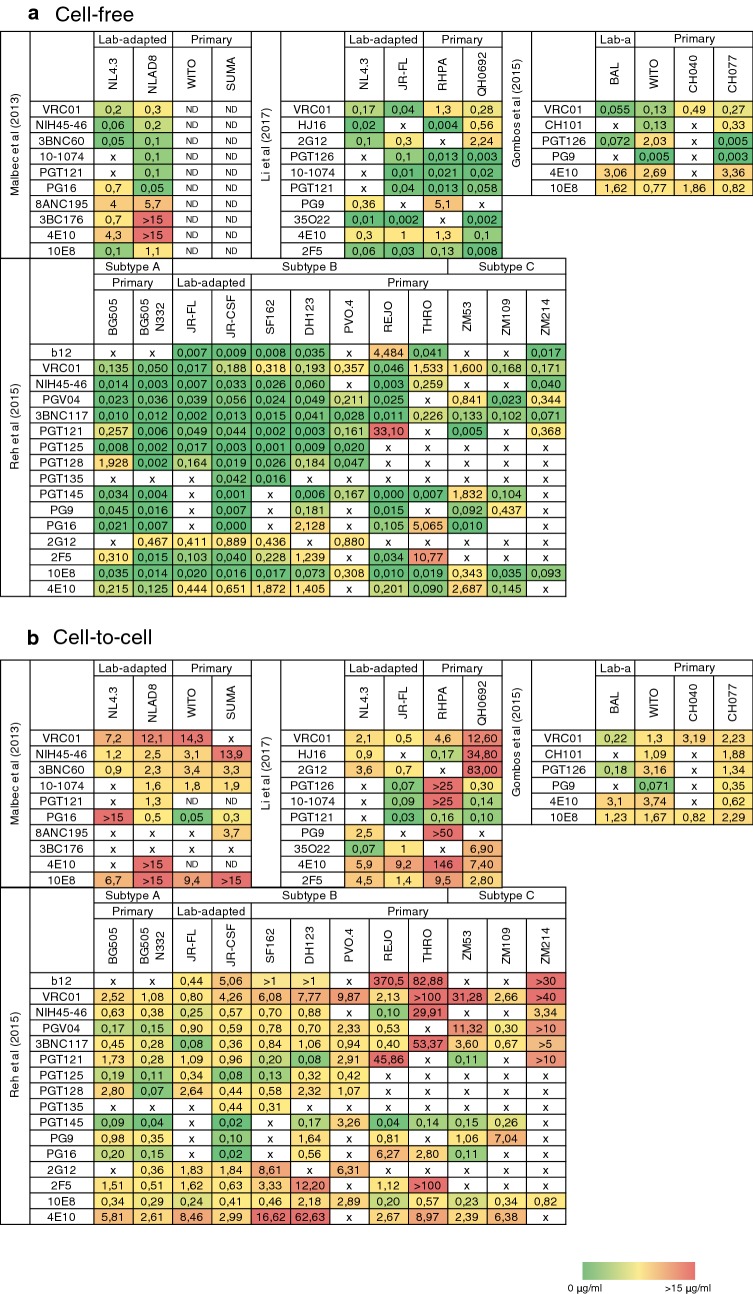
Neutralization potency of bNAbs against cell-free and cell-to-cell transmission of various viral strains. Cell-free (**a**) and cell-to-cell (**b**) neutralization IC50s of different bNAbs against several viral strains were compiled from the indicated studies (Malbec et al. [116]; Reh et al. [117], Gombos et al. [118], and Li et al. [119]). IC50s are color-coded with a heat map ranging from 0 (green) to 15 µg/ml and more (red). *x* not effective, no IC50 could be determined; *ND* not done; *Lab-a* lab-adapted

The development of second generation bNAbs allowed a more comprehensive examination of the role of antibodies during T cell-to-T cell transfer of HIV-1 (Fig. 2). The epitope targeted may influence the efficacy of a given antibody [115]. It has been shown that some CD4bs-directed antibodies were less potent neutralizers during cell-to-cell transmission than during cell-free transfer, with IC50s that were 10 times higher in intercellular systems [115]. Our laboratory tested the ability of 15 bNAbs targeting different Env epitopes to inhibit cell-to-cell transmission of both lab-adapted and Transmitted/Founder (T/F) HIV-1 strains [116]. We confirmed the relative neutralization resistance of cell-associated HIV-1. However, we identified bNAbs that were potent neutralizers of cell-associated virus in primary CD4^+^ T cells and pDCs. The most active bNAbs were targeting the CD4bs (NIH45-46 and 3BNC60) or the glycan/V3 loop (10-1074 and PGT121). They significantly decreased the formation of clusters and syncytia between uninfected and infected T cells, and the transfer of viral material through the VS. The efficacy of bNAbs against cell-associated HIV-1 was also dependent on the viral strain studied, indicating that the antibody breadth may be different against virions and infected cells. Another study analyzed the activity of 16 bNAbs during cell-free and cell-to-cell transmission of 11 viral strains [117]. Again, the neutralizing activity of bNAbs was generally decreased in cell-to-cell assays. Some bNAbs maintained a high level of inhibition against various viral strains, but no single bNAb was potent for all strains tested [117]. Combinating bNAbs may overcome this problem. For instance, a combination of PG9 and VRC01 demonstrated improved ability to neutralize cell-associated HIV-1 compared to individual antibodies [118]. Recently, a study focused on the maximum neutralization capacity of bNAbs during cell-to-cell transmission rather than on the IC50 [119]. During cell-to-cell transmission of two T/F strains, most of the tested bNAbs failed to reach 100% of neutralization, even at high concentrations. This phenomenon was not observed with two lab-adapted strains. This residual replication may allow the virus to keep spreading and may lead to the apparition of escape mutations. Whether the ability of primary HIV-1 isolates to spread by cell-to-cell transmission differs from lab-adapted strains, and how this may impair neutralization efficacy of bNAbs are still unresolved questions.

Antibodies interfere with HIV-1 cell-to-cell transmission through different mechanisms. For instance, b12 (a first generation anti-CD4bs antibody) inhibits the formation of the VS while 2F5 or 4E10 (anti-MPER) rather act later, by inhibiting viral fusion [114, 120]. Other bNAbs targeting the gp120, such as NIH45-46, 3BNC60, VRC01, 10-1074, or PGT121 also inhibit the formation of conjugates between infected and target CD4^+^ T cells [116]. Antibody efficacy varies depending on their time of addition in the co-culture [120]. For instance, b12 impairs VS formation, but does not disrupt an existing one [120]. Therefore, depending on the epitopes, bNAbs may either impair formation of cell conjugates and VS, transfer of viral material to target cells, or fusion.

### Inhibition of HIV-1 transfer from DCs and macrophages

HIV-1 transiting through a macrophage/T cell VS is inhibited by anti-gp120 bNAbs, but less sensitive to some anti-gp41 antibodies [68]. Early studies showed that neutralizing antibodies 2F5, 2G12 and b12 inhibited HIV-1 transfer from infected DCs to T cells without impairing the formation of the IS [121, 122]. The role of bNAbs on *trans*-infection is debated. 2F5-, 4E10- and 2G12-opsonized HIV-1 particles are captured more efficiently by DCs in a DC-SIGN-dependent manner, probably because DC-SIGN also binds IgG [123]. The particles recover their infectivity after internalization, probably due to antigen–antibody dissociation, leading to enhanced *trans*-infection. However, some bNAbs were also shown to inhibit infection or *trans*-infection from monocyte-derived or plasmacytoid dendritic cells to CD4^+^ T cells and vice versa [116, 124, 125]. In another study, gp120-targeting antibodies (b12, VRC01, PG16 and 2G12) had a higher IC50 against DC-associated virus, whereas anti-MPER 4E10 and 2F5 maintained their potency during DC-to-T cell transmission [126].

Therefore, some bNAbs inhibit *trans*-infection and transmission from DCs or macrophages to lymphocytes. Discrepancies have been reported for the same antibodies in different studies. These discrepant results likely depend on the DC subtype used, which may express different levels of molecules such as DC-SIGN, Siglec-1, or Env, at the surface or within intracellular compartments.

## Potential explanations for the increased resistance of cell-associated HIV-1 to neutralization by bNAbs

Different non-mutually exclusive mechanisms may account for the increased resistance of cell-to-cell HIV-1 transmission to bNAbs. They include steric hindrance at the VS, the MOI associated to this mode of viral propagation, the accessibility and conformation of Env at the cell surface, and the stability of Env-Ab complexes at the cell surface.

### Steric hindrance at the VS and in other cellular compartments

**Fig. 3:**
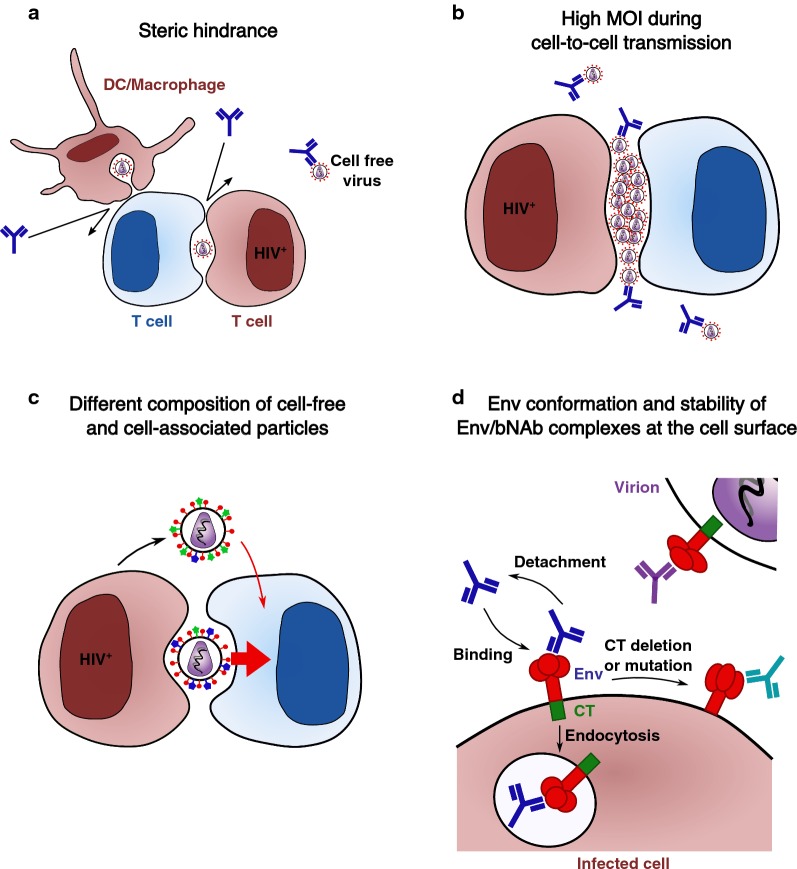
Potential mechanisms explaining the increased resistance of cell-associated HIV-1 to bNAbs-mediated neutralization. **a** bNAbs may poorly access virions present at the VS because of the physical proximity of donor and target cell membranes. **b** VS-mediated HIV-1 is associated with high MOIs. **c** Viruses budding at the VS may incorporate cellular proteins differently than cell-free virions, possibly leading to different susceptibilities to bNAbs. **d** Env conformation and stability of Env-bNAb complexes at the cell surface. Env conformation may be different at the surface of cell-free virus and at the plasma membrane. The stability of Env-bNAb complexes at the cell surface depends on the antibody and the viral strain. Donor cells are in brown and uninfected cells in blue

The VS involves a physical proximity of the membranes of donor and target T cells and may imply a low accessibility of bNAbs to the VS (Fig. 3a). However, some bNAbs like b12, NIH45-46 or 3BNC60 successfully accumulate at the VS between T cells [116, 120]. It will be of interest to determine whether access to the VS correlates with the inhibitory activity of each antibody. It is also possible that some antibodies bind to Env outside of the synapse, and will then be transported to the VS as a complex with their antigens. The virus may also be endocytosed after transmission through the VS [54], limiting the time frame of access of bNAbs. A llama antibody termed J3 is a potent neutralizer of cell-to-cell HIV-1 transmission [127]. The small size of the llama VHH compared to the human Fc may enable a better access to the VS. However, recombinant J3 with a human Fc display the same potency of neutralization against HIV-1 cell-to-cell transmission [127]. Thus, the size of the antibody does not seem to be a limiting factor in that case. The situation may be different in DCs or macrophages. A full-size 10E8 was less potent in these cells but 10E8 Fab, smaller in size, had more comparable neutralization IC50s during cell-free and cell-associated transmission [68]. This is consistent with the observation that bNAbs do not easily access virus contained within VCCs in macrophages [128]. This is also the case in DCs, where HIV-1 virions present in VCCs are protected from recognition by bNAbs, even if these compartments are connected to the extracellular milieu [89].

Thus, steric hindrance may impact neutralization of cell–cell transmission by some bNAbs and depends on the cell type and the antibody used. The most potent antibodies gain access to the VS and impair its function.

### Cell-to-cell HIV-1 transmission is associated with higher MOIs

The VS leads to an elevated concentration of viral particles in the synaptic cleft, which most likely increases the MOI during cell-to-cell transmission [37, 56–60] (Fig. 3b). Increased amounts of virus would then require more antibody, thus increasing IC50s. However, with some antibodies, differences in IC50s are still observed when cell-free and cell-associated viral inputs are normalized [126]. With the most potent bNAbs, such as 10-1074 and 3BNC117, the IC50s remain low in coculture systems [116, 117, 119]. Thus, differences between cell-to-cell and cell-free modes of transmission are not only a matter of quantity of transferred virus.

### Composition of cell-free particles and virions produced at the VS

Cell-free neutralization assays mostly use virus produced by transfection of 293T cells. Cell-to-cell assays generally rely on CD4 T cell lines or primary cells as the source of virus. Some HIV-1 strains are more susceptible to bNAbs when produced in 293T compared to primary cells [129]. This might be due to the content of cellular molecules in viral particles, as HIV-1 incorporates host membrane when budding. Thus, comparison between cell-free and cell-associated neutralization may be biased by the cell types in which the virus was produced. In addition, the composition of viral particles may vary at the VS (Fig. 3c). For example, HIV-1 virions can incorporate ICAM-1 that will increase infectivity, especially if the target cell expresses LFA-1 [130, 131]. ICAM-1-bearing virions are more resistant to neutralization by HIV-1-infected patients’ sera or neutralizing anti-gp120 antibodies [132]. Given that adhesion molecules accumulate at the VS, they could be more incorporated in viral particles budding at this site. The lipid component of VS-budding virions may be also different, since synapses are known to be enriched in rafts, and this may also impact sensitivity to neutralizing antibodies. Even though technically challenging, a characterization of the cellular composition of virions produced at the VS will give insights into the mechanisms underlying the resistance of cell-to-cell transmission to some bNAbs.

### Conformation and amount of Env at the cell surface and at the VS

The conformation and oligomerization states of Env are probably more heterogeneous at the plasma membrane than at the surface of virions, that contain a very limited number of Env trimers [133]. At the plasma membrane, a high amount of Env monomers and trimers, at different stages of maturation and glycosylation, are present. Local variations at membrane subdomains, depending on the subcellular environment or the presence of lipids and cellular proteins, may also modify Env epitope exposure. These different dynamic parameters impact the accessibility of cell-surface Env to bNAbs. For example, the engagement of Env by CD4 exposes epitopes targeted by non-neutralizing antibodies (nnAbs) [134, 135]. Since the creation of the VS involves interaction of Env with CD4, the conformation of Env at the synapse may be different from that at other regions of the membrane. We also reported that some antibodies, such as 8ANC195, which recognizes a gp120/41 bridging epitope and neutralizes cell-free virions, does not efficiently bind to infected cells [17], confirming the existence of different conformations of Env on virions and cells.

Viral proteins may also modify epitope accessibility. Nef and Vpu modify the levels of Env at the cell surface [136, 137] and Nef decreases Env susceptibility to anti-MPER antibodies [138]. The Env cytoplasmic tail (CT) also regulates the exposure of Env epitopes, through mechanisms that deserve further characterization [139, 140]. A CT truncation increases sensitivity to neutralization during cell-to-cell transmission with little effect on cell-free infection [110]. Some mutations in the CT inhibit cell-free infection more strongly than cell-to-cell transmission [141]. Mutations in the tyrosine-based sorting signal (YXXL) in the CT of two T/F strains modulate neutralization efficacy of b12, 10-1074 and PGT126 in cell-to-cell neutralization assays [119]. This YXXL motif regulates Env recycling from the plasma membrane. The engagement of Env in recycling pathways not only modulates the amount and stability of the viral protein at the surface, but may also impact epitope exposure (Fig. 3d).

### Stability of Env-bNAb complexes at the cell surface

The stability of Env-bNAb complexes at the cell surface most likely regulates the neutralization activity of bNAbs against donor cells. The half-life of Env-bNAb surface complexes depends on the antibody and the viral strain [17]. It varies from less than 30 min to more than 6 h [17]. These variations are likely due to the affinity of the antibody (association and dissociation rates), to antibody-induced Env internalization or shedding, or to other parameters that deserve further investigation. The natural recycling of Env at the plasma membrane or at the VS may also impact antibody efficacy during cell-to-cell transmission.

## Elimination of HIV-1-infected cells by bNAbs

Infected cells covered with potent bNAbs may be neutralized in their ability to transmit the virus, but may also become susceptible to antibody-mediated effector functions.

Antibodies are composed of a Fab region, responsible for antigen binding, and a Fc domain, recognized by Fc receptors expressed on immune cells. FcR engagement subsequently triggers various immune effector mechanisms (for a review, see [142]). For example, NK cells recruited by bNAbs kill HIV-1-infected cells through Antibody-Dependent Cellular Cytotoxicity (ADCC) [17, 143, 144]. Other Fc-dependent mechanisms include antibody–dependent cellular phagocytosis (ADCP) and activation of the complement pathway (reviewed in [142]). ADCC is mediated by bNAbs and nnAbs, depending on Env epitope accessibility at the cell surface [135, 145]. bNAbs require Fc-mediated immunity for optimal efficacy in vivo [146–148]. In humanized mice, nnAbs clear HIV-infected cells and impose selective pressure on the virus, as observed by mutation in Env [149]. However, primary strains are often poorly susceptible to nnAbs-mediated ADCC in vitro [135, 150]. HIV-1 propagation in vivo is the result of a balance between the rate of viral transmission and the clearance of infected cells. Thus, even if bNAbs do not totally neutralize viral cell-to-cell spread, Fc-mediated functions represent an additional mechanism of action of the antibodies against infected T cells. Whether these additional functions also impact DC/Macrophages-mediated *cis*- or *trans*-infection of CD4^+^ T cells remains poorly characterized.

## In vivo implications of the increased resistance of cell-to-cell transmission to bNAbs

Infectious body fluids such as blood, semen or breast milk contain both cell-free and cell-associated HIV-1 [151, 152]. In humans, comparing cell-free and cell-associated genetic signatures of the infecting partner’s virus to those of the founder virus in the recipient partner suggests that some infections are initiated by cell-associated virus [153]. Moreover, cell-associated Simian Immunodeficiency Virus (SIV) initiates infection in macaques [154, 155]. However, even though bNAbs combinations are efficient in murine and simian models, they were mostly tested in animals challenged with cell-free HIV-1 (reviewed in [19]). Recently, the effect of the anti-V3 antibody PGT121 was compared after cell-free or cell-associated Simian-Human Immunodeficiency Virus (SHIV) challenge in macaques [156]. PGT121 is efficient against cell-associated HIV-1 in vitro, requiring higher concentrations than during cell-free infection [116, 119]. PGT121 infusion protected all 6 animals challenged with cell-free SHIV. However, the antibody only protected 3 out the 6 animals challenged with infected cells. The 3 non-protected animals displayed 1- to 7-week delays in the onset of viremia. This delay correlated with PGT121 serum concentrations. Thus, PGT121 was only partially effective against cell-associated SHIV challenge in macaques. This may be due to an “occult” infection which triggered viral spread when bNAbs levels waned, or to the transfer of latently infected cell that reactivated late after the challenge. These results highlight the need for high and sustained concentrations of antibodies to confer resistance to challenge with infected cells. In this macaque model, a high dose of cell-associated SHIV was used as a challenge. Humans probably receive a lower level of infectious challenge during natural contamination. Future trials of bNAbs will be of great interest to assess their prophylactic efficacy in humans.

Noteworthy, the main issue of using single bNAbs in vivo is the rapid occurrence of escape mutations [23–26]. Mathematical modelling suggested that escape mutations to bNAbs are more likely to happen during cell-to-cell transmission than during cell-free infection [117]. Again, current and future clinical trials using combination of bNAbs will be instrumental in determining whether this immunotherapy is counteracting the different modes of HIV-1 spread in humans.

## Conclusion

HIV-1 cell-to-cell transfer has been extensively characterized in cell culture systems. In vivo experiments confirmed the contribution of this mechanism during viral spread. Conventional antiretroviral drugs efficiently inhibit cell-free and cell-associated viral transmission [157] but the impact of bNAbs on intercellular viral spread may be less marked. There are important mechanistic differences depending on antibodies, viral strains, and the nature of donor and recipient cells. The most potent bNAbs, that stably bind to infected cells and impair CD4/Env interaction or viral fusion, efficiently inhibit cell-to-cell transfer. These bNAbs display transient therapeutic efficacy in humans. In addition to neutralization, bNAbs trigger the destruction of infected cells. Future basic and clinical studies will help determining whether the targeting of infected cells by combinations of bNAbs with long half-lives and increased potency are a promising approach to the prevention, treatment, and possibly cure of HIV-1 infection.
